# Plasma Extracellular Vesicle α-Synuclein Level in Patients with Parkinson’s Disease

**DOI:** 10.3390/biom11050744

**Published:** 2021-05-17

**Authors:** Chen-Chih Chung, Lung Chan, Jia-Hung Chen, Yi-Chieh Hung, Chien-Tai Hong

**Affiliations:** 1 Department of Neurology, Taipei Medical University–Shuang Ho Hospital, New Taipei City 23561, Taiwan; 10670@s.tmu.edu.tw (C.-C.C.); cjustinmd@gmail.com (L.C.); gary.320@hotmail.com (J.-H.C.); 2 Department of Neurology, School of Medicine, College of Medicine, Taipei Medical University, Taipei 11031, Taiwan; 3 Graduate Institute of Biomedical Informatics, Taipei Medical University, Taipei 11031, Taiwan; 4 Department of Neurosurgery, Department of Surgery, Chi-Mei Medical Center, Tainan 71004, Taiwan; 5 Department of Recreation and Healthcare Management, Chia Nan University of Pharmacy and Science, Tainan 71710, Taiwan

**Keywords:** extracellular vesicles, Parkinson’s disease, akinetic-rigidity, α-synuclein

## Abstract

Background: The most established pathognomonic protein of Parkinson’s disease (PD), α-synuclein, is extensively investigated for disease diagnosis and prognosis; however, investigations into whether the free form of α-synuclein in the blood functions as a PD biomarker have not been fruitful. Extracellular vesicles (EVs) secreted from cells and present in blood transport molecules are novel platforms for biomarker identification. In blood EVs, α-synuclein originates predominantly from the brain without the interference of the blood–brain barrier. The present study investigated the role of plasma EV-borne α-synuclein as a biomarker of PD. Methods: Patients with mild to moderate stages of PD (*n* = 116) and individuals without PD (*n* = 46) were recruited to serve as the PD study group and the control group, respectively. Plasma EVs were isolated, and immunomagnetic reduction–based immunoassay was used to assess EV α-synuclein levels. Conventional statistical analysis was performed using SPSS 25.0, and *p* < 0.05 was considered significant. Results: Compared with controls, we observed significantly lower plasma EV α-synuclein levels in the patients with PD (PD: 56.0 ± 3.7 fg/mL vs. control: 74.5 ± 4.3 fg/mL, *p* = 0.009), and the significance remained after adjustment for age and sex. Plasma EV α-synuclein levels in the patients with PD did not correlate with age, disease duration, Part I and II scores of the Unified Parkinson’s Disease Rating Scale (UPDRS), or the Mini-Mental State Examination scores. However, such levels were significantly correlated with UPDRS Part III score, which assesses motor dysfunction. Furthermore, the severity of akinetic-rigidity symptoms, but not tremor, was inversely associated with plasma EV α-synuclein level. Conclusion: Plasma EV α-synuclein was significantly different between the control and PD group and was associated with akinetic-rigidity symptom severity in patients with PD. This study corroborates the possible diagnostic and subtyping roles of plasma EV α-synuclein in patients with PD, and it further provides a basis for this protein’s clinical relevance and feasibility as a PD biomarker.

## 1. Introduction

Parkinson’s disease (PD) is the second most common neurodegenerative disease [1]. The pathological hallmark of PD is the formation of Lewy bodies inside the midbrain dopaminergic neurons. These bodies are composed of the aberrant accumulation of pathognomonic proteins such as tau and β-amyloid (Aβ) and especially α-synuclein [2]. Because of the difficulty in obtaining brain tissue, body fluid proteins are instead extensively investigated as biomarkers for the diagnosis and progression prediction of PD. A reduction of α-synuclein in the cerebrospinal fluid (CSF) has consistently been observed in patients with PD [3].

Lumbar puncture, the sole manner in which CSF is obtained, is invasive and unpopular because of the associated adverse effects, including headache and back pain [4]. Peripheral blood is a more acceptable source of body fluid for the investigation of circulating biomarkers in clinical practice. However, the dynamism of α-synuclein in the blood, especially secondary to diet, lifestyle, and physical activity, results in substantial variability. Many studies have investigated PD-pathognomonic proteins in the blood or serum as disease biomarkers, but controversial and inconclusive results have been obtained [5].

A preferred alternative is to identify other blood components that are stable and accurately reflect cellular and systemic conditions. Extracellular vesicles (EVs) are particles secreted by cells that carry abundant proteins and nucleotides [6]. In the last decade, exosomes, which are a type of EV with a diameter of 30–100 nm, have attracted increased research attention [6]. Owing to their lipid-layered outer membrane, exosomes can cross the blood–brain barrier (BBB) and remain stable in the blood for a long period [7]. This high structural stability prevents the degradation of circulating biomarkers and reflects the intraneuronal condition in the periphery. EV proteins in the blood have been used as biomarkers for multiple diseases, including cancer [8], cardiovascular diseases [9], and Alzheimer disease (AD) [10].

EV-borne biomarkers, including proteins [11,12,13] and microRNA [14,15], have also been extensively examined in the context of PD. Cellular α-synuclein is transported to exosomes via the endosome pathway [16]. Several studies have investigated the α-synuclein concentration of EVs from different origins, including blood [17,18,19,20], CSF [21], and saliva [22]. However, the results were controversial. Considering that α-synuclein may be associated with PD diagnosis, disease progression, and therapeutic response, we hypothesized that α-synuclein transported by plasma EVs could serve as a biomarker for the diagnosis and subtype classification of PD.

## 2. Materials and Methods

### 2.1. Study Participants

One hundred and sixty-two participants (116 in the PD group and 46 in the control group) were enrolled in this study from November 2017 to September 2019 at the Department of Neurology, Shuang Ho Hospital. Diagnoses of PD were based on the UK Parkinson’s Disease Society Brain Bank Diagnostic Criteria [23]. Only patients with mild to moderate PD, defined as Stage I to III PD according to the Hoehn and Yahr scale, were included in the PD group. The control individuals were free from known neurodegenerative, psychiatric, and major systemic diseases such as malignant neoplasm and chronic kidney disease, and they received regular outpatient follow-ups for chronic neurological conditions (transient ischemic attack, dizziness, headache, and diabetic polyneuropathy). People with a Mini-Mental State Examination (MMSE) score less than 26 were excluded from the control group. This study was approved by the Joint Institutional Review Board of Taipei Medical University (approval no. N201609017, Approval date: 25/Nov/2016, and N201801043, Approval date: 23/Feb/2018), and all study participants signed informed consent forms prior to participation.

### 2.2. Clinical Assessments

All participants were interviewed to obtain baseline demographic data. The cognitive function of all study participants was assessed by trained nurses using the Taiwanese versions of the MMSE. All patients with PD were evaluated using Parts I, II, and III of the Unified Parkinson’s Disease Rating Scale (UPDRS) during an outpatient visit. The time between the most recent dose of anti-PD medication and the application of the UPDRS Part III was not recorded; patients with PD were assumed to be in their “on” time. The akinetic-rigidity (AR) score and tremor score were obtained from the relevant items in the UPDRS Part III and calculated as previously described [24].

### 2.3. Plasma EV Isolation and Validation

For the isolation of plasma EVs, venous blood samples were collected from all study participants following the instructions of the International Society for Extracellular Vesicles [25]. Whole blood was centrifuged at 13,000 × *g* for 20 min to isolate the plasma. Next, 1 mL of plasma from each participant was passed through an exoEasy Maxi kit (Qiagen, Venlo, NL, Cat. #76064) for exosome isolation following the manufacturer’s instructions.

Isolated EVs were validated by detecting tetraspanins (CD9, CD81, and CD63), Inner components of EVs (tumor susceptibility gene 101), the negative segments of mitochondrial protein (cytochrome c), and their sizes were determined through nanoparticle tracking with a peak of approximately 100 nM. Details on the validation were described in our previous studies involving this PD cohort [12,13].

### 2.4. Immunomagnetic Reduction Assay

Assessment of the targeted pathognomonic proteins in the blood through immunomagnetic reduction (IMR) assay has been discussed and validated in the literature [26,27,28]. The principle behind the immunomagnetic reduction (IMR) assay is the measurement of the alternating-current magnetic susceptibility of magnetic nanoparticles coated with surfactants (dextran) and bioprobes such as antibodies. The mean hydrodynamic diameter of the antibody-functionalized magnetic nanoparticles was approximately 55 nm. The concentration of each reagent was approximately 10 mg Fe/mL. For α-synuclein IMR assays, 80 μL of reagent was mixed with 40 μL of the plasma EV sample. After the reagent and plasma sample were mixed, each mixture was placed into a superconducting quantum interference device (SQUID)-based magnetosusceptometer (XacPro-S, MagQu Co., Ltd., New Taipei City, Taiwan). The analyzer applied alternative magnetic fields to oscillate each magnetic nanoparticle in PBS solution. Due to nanoparticle oscillation, the reagent generated alternative magnetic signals. The resultant strength of the alternative signals depended on the oscillating efficiency of magnetic nanoparticles. All recopies of IMR measurements were optimized to maximize the nanoparticle oscillation unbounded with target biomolecules. Thus, we had a maximized resultant alternative magnetic signal before the association between nanoparticles and target biomolecules. After the association between the magnetic nanoparticles and target biomolecules, the bound nanoparticles became larger, which led to the suppression of the oscillation efficiency. Hence, the reduction in the alternative magnetic signals of the reagent resulted. As a sample had a higher concentration of target biomolecules, more magnetic nanoparticles in the reagent associate were bound with target biomolecules. The reduction in the alternative magnetic signals of the reagent was enhanced. Therefore, the concentrations of target biomolecules, i.e., α-synuclein in this work, were determined according to the reduction in the alternative magnetic signals of the reagent [29]. The reduction in the alternative magnetic signals of the reagent due to the association between magnetic nanoparticles and target biomolecules is referred to as the IMR signal hereafter. The IMR signal thus reflected the concentration of the targeted protein. Assays were replicated in duplicate for each biomarker and sample. The reported concentration of each PD-pathognomonic protein was the mean value of replicated measurements. The α-synuclein (MF-ASC-0060) and reagents were conjugated with specific antibodies against α-synuclein (sc-12767; Santa Cruz, CA, USA) proteins, and the Ser129 phosphorylated α-synuclein (MF-PS1-0060) reagents were conjugated with specific antibodies against Ser129 phosphorylated α-synuclein (825701; Biolegend, San Diego, CA, USA) proteins. According to the instructions from MagQu Co., Ltd., the assay limit of detection was 1.39 fg/mL for α-synuclein and 0.072 fg/mL for Ser129 phosphorylated α-synuclein.

### 2.5. Statistical Analysis

All statistical analyses, except for the generation of artificial neural network models, were performed using IBM SPSS for Windows, version 22 (IBM Corp., Armonk, NY, USA). A chi-squared test was used to compare the sex distribution between the PD group and the control group. A nonparametric Mann–Whitney U test was used to compare the plasma EV levels of α-synuclein as well as other continuous variables between the PD group and the control group, and Spearman’s rank correlation was used to investigate the correlation between the plasma EV α-synuclein level and clinical factors. Multivariable logistic regression was employed to investigate the association between plasma EV α-synuclein levels and the motor scores of patients with PD adjusted for age, sex, and disease duration. A *p*-value of <0.05 was considered statistically significant.

## 3. Results

As presented in Table 1, the baseline data indicate no differences between the age and sex of patients with PD and the control group. Because people with an MMSE score of <26 were excluded from the control group, a significant difference in cognitive function was recorded, as determined through a comparison of the MMSE scores of patients with PD and the control group. The mean PD duration was 2.82 ± 2.48 years, and the mean UPDRS scores in Part I, II, and III were 2.48 ± 2.00, 7.92 ± 5.82, and 22.48 ± 9.85, respectively.

**Figure 1:**
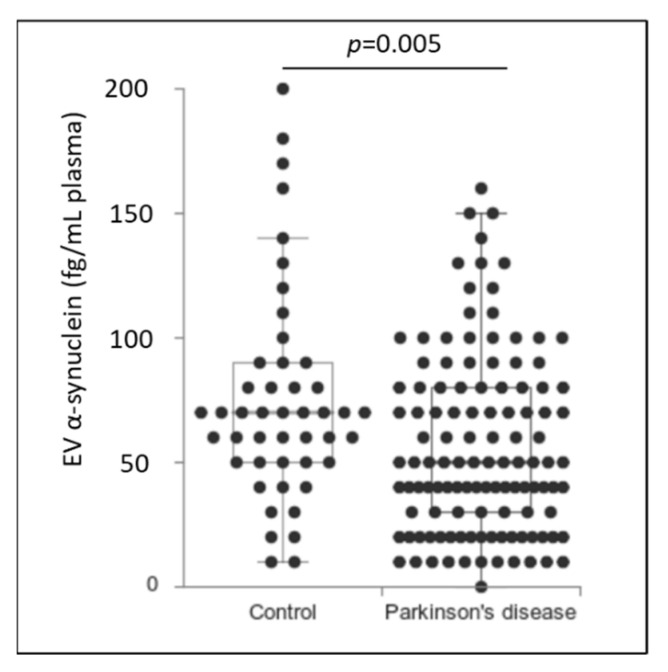
Plasma extracellular vesicle α-synuclein in control group participants and patients with Parkinson’s disease. Box and dot plots showing the differential protein level of plasma EV α-synuclein in control participants and patients with Parkinson’s disease. Data are illustrated as raw data and box plots with median, first quartile, third quartile, and 5th/95th percentile values.

Patients with PD had significantly lower levels of α-synuclein in plasma EVs than the control group did (PD: 56.0 ± 3.7 fg/mL vs. control: 74.5 ± 4.3 fg/mL, *p* = 0.005) (Figure 1). The negative association of plasma EV α-synuclein with a diagnosis of PD remained significant after adjustment for age and sex (*p* = 0.009, 95% confidential interval (CI) = −4.078 to −0.583 (Table 2). The effect of using plasma EV α-synuclein on the diagnosis of PD was modest, and the receiver operating characteristic curve yielded the area under the curve (AUC) of 0.631. After setting up the cut-off value of plasma EV α-synuclein at 196 fg/mL, the sensitivity of the PD diagnosis was 50% with the specificity of 76% (Supplementary Figure S1).

**Figure 2:**
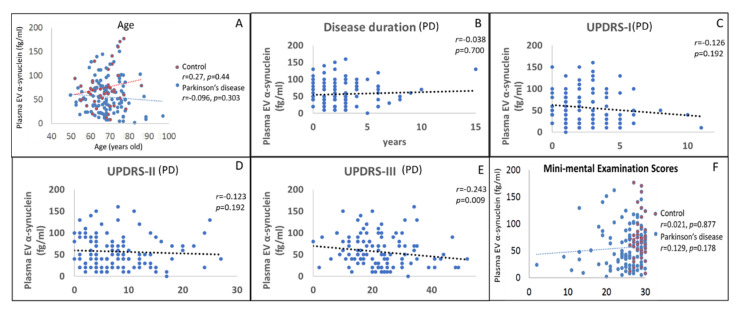
The crude correlation of clinical factors with the levels of plasma extracellular vesicle (EV) α-synuclein among patients with Parkinson’s disease (PD) and controls. The association of plasma EV α-synuclein with (**A**) age, (**B**) disease duration, (**C**) Unified Parkinson’s Disease Rating Scale Part I (UPDRS-I) score, (**D**) UPDRS Part II score, (**E**) UPDRS Part III score, and (**F**) Mini-Mental State Examination score.

We further investigated the correlation between plasma EV α-synuclein and the demographic information of patients and their PD severity. The plasma EV α-synuclein was not correlated with age and disease duration. Regarding disease severity, plasma EV α-synuclein was significantly and negatively correlated (r = −0.243, *p* = 0.009) with UPDRS Part III score (motor symptom severity). Plasma EV α-synuclein levels were not significantly correlated with UPDRS Part I (mental), UPDRS Part II (daily activity), or MMSE scores (Figure 2). Among 27 younger PD patients in our study (age less than 70 years), plasma EV serine 129 phosphorylated α-synuclein, the toxic form of α-synuclein, was assessed by the IMR method as well. There was a significant positive correlation (β = 0.403, *p* = 0.037) (Supplementary Figure S2) between plasma EV total and serine 129 phosphorylated α-synuclein. However, because of the small case number, the plasma EV serine 129 phosphorylated α-synuclein did not achieve the significant correlation with the severity of motor symptoms.

We observed that after adjustment for age, sex, and disease duration, plasma EV α-synuclein levels had no association with UPDRS Part I, II, or III scores. However, if the UPDRS III scores were segregated into tremor and AR scores, plasma EV α-synuclein level was negatively correlated with akinetic-rigidity severity after adjustment for age, sex, and disease duration (*p* = 0.025, 95% CI = −4.86 to −0.37) (Table 3).

## 4. Discussion

The present study demonstrated that the plasma EV α-synuclein levels were significantly lower in patients with PD and negatively correlated with the severity of AR subtype motor symptoms. We posit that the study of plasma EV α-synuclein may provide mechanistic insight into the correlations between PD-pathognomonic protein expression variability, PD pathogenesis, and the associated distinct clinical presentation of motor symptoms in patients with PD.

EVs are secreted from most human cells and facilitate remote cell-to-cell communication. Evidence implicating plasma EV concentration in disease pathogenesis continues to accrue, as demonstrated by recent studies on AD and insulin resistance [30,31]. The EV’s cargo of intracellular proteins often reflects a cellular pathology; the engulfment of such proteins by EVs could represent cytoplasm mimicry. The most widely studied pathognomonic protein related to PD is α-synuclein. However, the accumulation of other proteins, such as β-amyloid and tau, have also been observed in postmortem examinations of the brains of people with PD [32]. The detection of these proteins in the CSF is promising for diagnosis and outcome prediction; however, the use of lumbar puncture to obtain CSF is not common practice for patients with suspected PD because of its moderate invasiveness, inconvenience, and possible side effects. Plasma EVs are an ideal alternative platform for PD-related biomarker studies, particularly because the structural stability of EVs precludes the degradation of transported proteins in circulation [6]. This study demonstrated a functional association between plasma EV α-synuclein level and the motor symptoms of patients with PD. These results indicate that plasma EV α-synuclein is closely associated with the presence of motor symptoms in patients with PD; this association is reconcilable with a contemporary clinical diagnosis of PD, and thus, plasma EV α-synuclein can serve as one of the biomarker panels for diagnosis of PD.

The present study revealed an association between plasma EV α-synuclein level and the severity of the AR subtype motor symptoms in patients with PD. PD is a heterogeneous disease with two major motor subtypes, namely tremor-predominant (TD) PD and the AR subtype of PD [33]. The AR subtype is characterized by rapid progression, comorbidity with cognitive dysfunction, and an early loss of life independence [34] The aggregation of α-synuclein in Lewy bodies is less prominent in the TD subtype of PD than in the AR subtype [35,36]. In this case, plasma EV α-synuclein level may be associated with a Lewy body pathology in individuals with PD. A reduction in plasma EV α-synuclein may result from the sequestration of the toxic aggregated form of α-synuclein in the neurons of patients with PD, which affects the cargo of the α-synuclein monomer into EVs for secretion [5].

EV α-synuclein is transported by exosomes to the endosome pathway [16]. Endosomes transporting α-synuclein transition into multivesicular bodies and fuse with the plasma membrane for secretion as EV cargoes; this process is regulated by variations in intracellular calcium concentration [37]. Several studies have investigated the α-synuclein concentrations in EVs from different origins, including blood [17,18,19,20], CSF [21], and saliva [22]. Although the accumulation of α-synuclein in the substantia nigra is a well-documented pathological feature of PD, this elevated α-synuclein level is not universal; α-synuclein levels in EVs and the CSF of patients with PD are relatively low [38]. The sequestration of the α-synuclein into fibrillar aggregates in Lewy bodies, and the increase in the uptake of α-synuclein in neurons were the possible rationales for the reduction of α-synuclein in CSF and plasma EV, as well [39,40]. The result of the present study demonstrated the reduction of plasma EV total α-synuclein in PD patients, which was not compatible with previous studies demonstrating the elevation of α-synuclein in the blood neuron-derived exosomes from PD patients [17,18]. The distinction may result from the difference in the EV isolation, type of EV selection, and the method of α-synuclein detection. This inconsistency was found in the previous studies measuring the blood free form α-synuclein in PD patients as well, which was reviewed by Bougea et. al. [41], and more studies are warranted to achieve a solid conclusion. We are cognizant of the probable erythrocyte origin of EVs bearing α-synuclein, which constitutes a possible source of bias in data interpretation. However, according to the previous literature almost all plasma EV α-synuclein proteins are neuron-derived [18].

This study revealed a significant reduction in plasma EV α-synuclein levels in patients with PD. In addition, plasma EV α-synuclein level was significantly and negatively correlated with the severity of AR syndromes in patients with PD. Our findings are corroborated by evidence of a lower central nervous system-derived exosomal α-synuclein level in the blood of patients with the AR subtype of PD compared with the TD subtype [19]. From our results, we posit a probable association between lower plasma EV α-synuclein level and a more severe Lewy body brain pathology (the AR subtype). Plasma EV α-synuclein may serve as both a disease subtyping biomarker and a treatment response parameter for the upcoming vaccination and monoclonal antibody treatment targeting the elimination of intracranial α-synuclein aggregation.

This study did not indicate a correlation between plasma EV α-synuclein and cognition. One possible explanation is that PD-associated cognitive decline and dementia result from the progression of α-synuclein pathology, wherein Lewy bodies travel from the brainstem to the cortex [42]. However, regarding cognitive impairment in patients with PD, other brain pathologies as comorbidities, including amyloid, neurofibrillary tangle, or vascular insults, play significant roles as well [2]. Further multicenter studies with a large sample size are warranted to observe changes in PD-pathognomonic proteins, namely α-synuclein, tau, and Aβ1–_42_ proteins, in plasma EVs and determine their association with cognitive impairment in patients in the early stages of PD.

This study had some limitations, including the use of total plasma EVs instead of solely neuron-derived EVs. However, in blood EVs, α-synuclein originated predominantly from the nervous system, including central and peripheral nervous systems. [18], making the future clinical application of total plasma EV isolation feasible. Second, the present study assessed α-synuclein in total plasma EVs but not the highly toxic phosphorylated or oligomeric α-synuclein. Third, some of the patients with early-stage PD had low MMSE scores because of their low education level. The extension of the mandatory education period from 6 to 9 years in Taiwan in 1968 means that people older than 64 years were most likely to have had 6 years of basic education, thus affecting their cognitive capabilities in test performance. Moreover, unlike the healthy controls used in other studies, the present study used people of relative age with no signs of PD for the control group, and they attended regular outpatient clinic follow-ups for other underlying illnesses, including hypertension, diabetes, and hyperlipidemia. Although individuals in the control group may not have had established diagnoses of memory decline, it is not inconceivable that some may have had subjective or cognitive impairment and prodromes of neurodegeneration.

## 5. Conclusions

We demonstrated that patients with PD exhibited significantly lower levels of plasma EV α-synuclein than those in the control group did; such low levels are associated with motor symptom severity, especially of AR symptoms, in patients with PD. The present study lays the foundation for a future large-cohort, multicenter, longitudinal investigation of the correlation between the plasma EV α-synuclein and multiple aspects of disease subtyping and progression.

## Figures and Tables

**Table 1:** Demographic data for patients with Parkinson’s disease and control group participants.

	Control	PD	*p*-Value
Number of patients	46	116	-
Age (years)	67.04 ± 7.04	69.66 ± 8.41	0.06
Female	28	54	0.12
Disease duration (years)	-	2.82 ± 2.48	-
MMSE	28.41 ± 1.24	24.17 ± 6.36	<0.001
UPDRS Part I		2.48 ± 2.00	-
UPDRS Part II		7.92 ± 5.82	-

MMSE, Mini-Mental State Examination; MoCA, Montreal Cognitive Assessment; UPDRS, Unified Parkinson’s Disease Rating Scale.

**Table 2:** Multivariable regression model representing the association between serum extracellular vesicle (EV) α-synuclein levels and a diagnosis of Parkinson’s disease.

	Std.β	*p*-Value	95% CI
EV α-synuclein	−0.202	0.009	−4.078 to −0.583
Age	0.122	0.117	−0.002 to 0.015
Sex	0.099	0.202	−0.048 to 0.226

Std, standard; β, coefficient; CI, confidence interval.

**Table 3:** Association between serum extracellular vesicle α-synuclein level and each Unified Parkinson’s Disease Rating Scale (UPDRS) subtype after adjustment for age, sex, and disease duration.

	Std.β	*p*-Value	95% CI
UPDRS Part I	−0.139	0.159	−18.91 to 3.14
UPDRS Part II	−0.065	0.505	−42.45 to 21.05
UPDRS Part III	−0.160	0.097	−93.9 to 7.98
Akinetic-rigidity score	−0.213	0.025 *	−4.86 to −0.37
Tremor score	−0.012	0.904	−0.51 to 0.45

Std, standard; β, coefficient; CI, confidence interval; * *p* < 0.05.

## Data Availability

Please contact the corresponding authors (Y.-C.H. and C.-T.H.). The use of data and materials requires the permission of the TMU-JIRB.

## Supplementary Materials

The following are available online at https://www.mdpi.com/article/10.3390/biom11050744/s1, Figure S1: The receiver operating characteristic (ROC) curve of the diagnosis of Parkinson’s disease by the plasma extracellular vesicle α-synuclein. Figure S2: The correlation between plasma extracellular vesicle total and ser129 phosphorylated α-synuclein.
