# Supplementary material for: Plasma Extracellular Vesicle α-Synuclein Level in Patients with Parkinson’s Disease
Source: Biomolecules. 2021 May 17;11(5):744. doi: 10.3390/biom11050744 (PMC8155846; doi:10.3390/biom11050744)

# Supplementary information

Supplementary Figure 1. The receiver operating characteristic (ROC) curve of the diagnosis of Parkinson's disease by the plasma extracellular vesicle α-synuclein

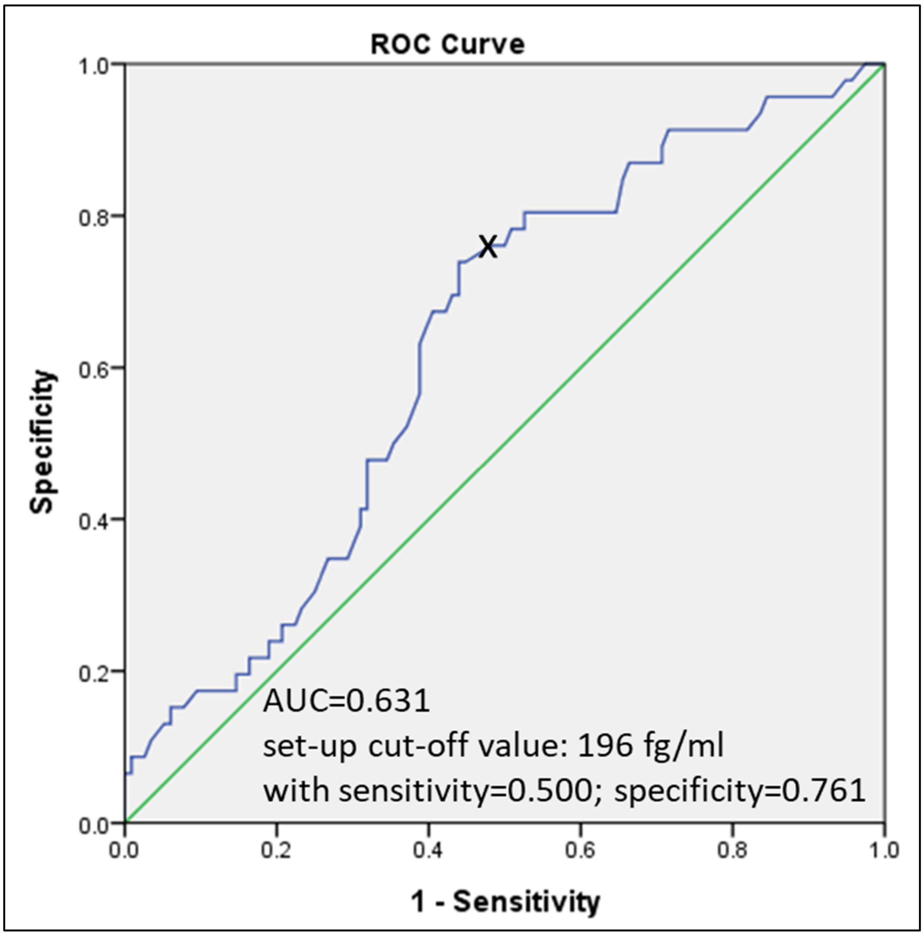

Supplementary Figure 2. The correlation between plasma extracellular vesicle total and ser129 phosphorylated α-synuclein.

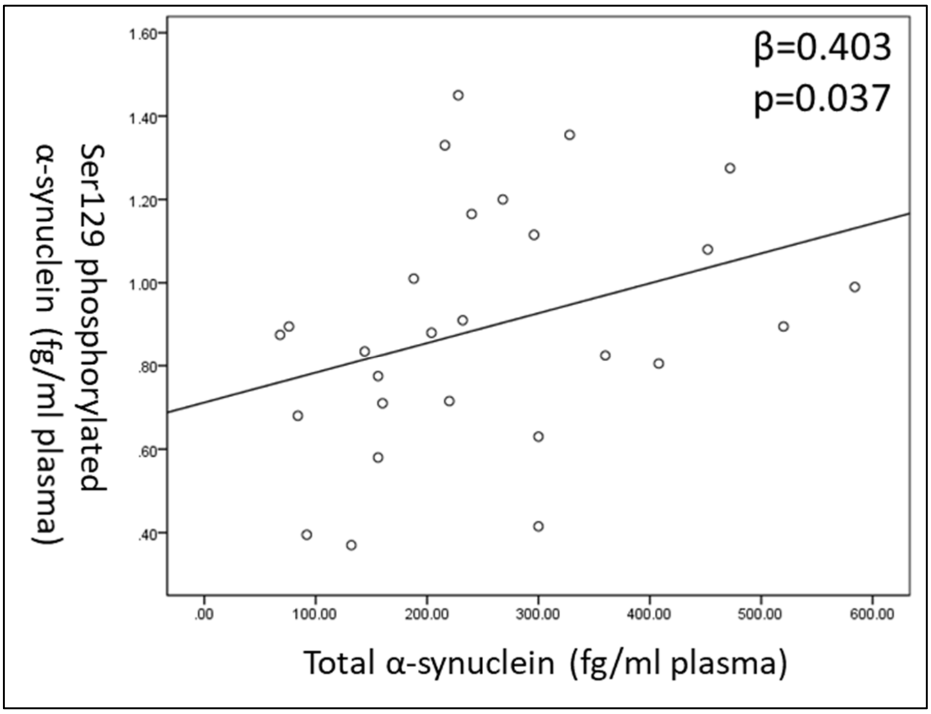

Supplement: Supplementary file 1 [file biomolecules-11-00744-s001.zip › biomolecules-1192799-supplementary.pdf]
